# Traumatic Tympanic Membrane Perforation in Children in the Emergency Department: Comparison of Spontaneous Closure and Paper Patch

**DOI:** 10.7759/cureus.7697

**Published:** 2020-04-16

**Authors:** Serkan Cayir, Huseyin Mutlu

**Affiliations:** 1 Otolaryngology, Aksaray Education and Research Hospital, Aksaray University, Aksaray, TUR; 2 Emergency Medicine, Faculty of Medicine, Aksaray University, Aksaray, TUR

**Keywords:** tympanic membrane, traumatic perforation, paper patch, spontaneous closure

## Abstract

Objective

There are different clinical approaches for traumatic tympanic membrane perforation in the pediatric age group. The purpose of this study was to compare spontaneous recovery and the paper patch procedure and to analyze the factors that play a role in recovery.

Materials and methods

Pediatric patients who were admitted to the Emergency Department between January 2014 and June 2019 due to traumatic tympanic membrane perforation were investigated. Among these patients, medical records of cases followed by the otorhinolaryngology clinic for spontaneous closure or paper patch procedure were retrospectively examined. Medical records of a total number of 71 patients aged 2-16 years (33 females, 38 males) were analyzed.

Results

The overall closure rate was 89.75% (64/71). Although there was no difference between the groups of small- and medium-size perforations in terms of closure rates, the closure rate in large perforations was 90.9% in the paper patch group and 63.6% in the spontaneous closure group, and the difference between these two groups was statistically significant (p < 0.05). Additionally, the closure rate in the paper patch group (91.6%) was found to be significantly higher than that in the spontaneous closure group (58.3%) in the presence of a perforation contacting the malleus (p > 0.05).

Conclusion

Both procedures can be employed in pediatric cases of traumatic tympanic membrane perforation with high success rates. However, in case of a large perforation or a perforation contacting the malleus that is detected in the Emergency Department, it is necessary to refer the patients to the otorhinolaryngology clinic immediately for paper patch procedure instead of following up the patients for spontaneous closure.

## Introduction

Tympanic membrane perforations (TMPs) in children occur owing to various reasons, and the most common reasons include blunt trauma to the ear, barotrauma, and foreign objects. The tympanic membrane is highly sensitive to sudden changes in pressure in the external auditory canal and may get easily affected by these changes and get damaged. These perforations that occur are generally prone to spontaneous closure; however, the perforation size and possibility of spontaneous recovery are negatively correlated, and large perforations need longer recovery time [1]. Epithelial regeneration areas are present in the proximities of the annulus and the manubrium of the malleus, and perforations heal through epithelial migration from both directions [2]. There are two separate categorizations of this migration. Epithelial migration that occurs toward the perforation center on a flat plane is described as centripetal migration, whereas migration that occurs upward and toward the distal end of perforation is described as outward migration [3].

The clinically practical approach is mostly conservative and indicates that the ear should be protected from infection and that the external auditory canal should be kept clean and dry. A follow-up duration of three to four weeks following perforation is sufficient for spontaneous closure in cases of small perforations; however, various surgical procedures such as myringoplasty might be necessary in some of the cases in which perforation closure does not occur at the end of this period [4]. Signs of spontaneous closure should therefore be carefully monitored at the perforation site during follow-up. Materials such as cigarette paper, fat, gelatin film, and silk can be used in the paper patch procedure, which can be easily performed and does not require hospitalization [5,6]. The most common and cost-effective material used in clinical practice is cigarette paper.

There are limited studies on TMP in children in the relevant literature. Therefore, this study intended to investigate spontaneous closure and the associated factors in children with TMP aged less than 18 years and to compare these results with those of the paper patch procedure. The patients were also compared in terms of their recovery times.

## Materials and methods

Medical records of 71 patients who presented to the Emergency Department between January 2014 and June 2019 and were followed up owing to traumatic TMP by the otorhinolaryngology clinic were retrospectively analyzed. Their demographic data, causes and characteristics of perforation, treatment methods, and follow-up periods were analyzed. Patients who presented to the hospital after more than two weeks, those who had a history of a perforated eardrum, and those who had a history of ventilation tube or eardrum surgery were excluded from study.

The initial diagnoses of all patients were made by an emergency medicine specialist or an attending otorhinolaryngology physician, and the patients were then followed up by the otorhinolaryngology clinic. Eardrum perforations of <25% were classified as small perforations, those of 25%-50% as medium perforations, and those of >50% as large perforations. Some of the patients were followed up for spontaneous recovery, whereas the paper patch procedure using cigarette paper was performed in other patients, and the patients were provided with detailed information about both procedures. The paper patch procedure was performed in patients selected for this procedure under sedoanalgesia (midazolam and ketamine), and a piece of cigarette paper 1.5 times larger than the perforation itself was placed as an overlay after de-epithelization of the edges of the perforation with trichloroacetic acid. Those who had ear discharge in both groups were administered antibiotic treatment, whereas the rest were not. The patients in the spontaneous closure group were followed up until spontaneous recovery occurred. The follow-up period of the patients lasted three months, and their perforation sites were assessed at outpatient clinic visits every two weeks.

Statistical analysis

The study data were statistically analyzed using SPSS, Version 21 (IBM Corp., Armonk, NY, USA). Data were represented as mean ± standard deviation and as proportion (%). Chi-square test was used to compare the categorical data between the two groups. The Mann-Whitney U test was used for non-parametric variables, whereas paired sample t-test was used for parametric variables. The results with a p-value of <0.05 were considered statistically significant. The characteristics of the patients in both groups are presented in Table 1.

 

**Table 1:** Characteristics of patients

	Group 1: Paper Patch ( N = 38)	Group 2: Spontaneous Closure ( N = 33)	p-Value
Age (mean)	13.4 ± 1.19	13.8 ± 1.33	p>0.05
Gender (female:male)	17:21	16:17	p>0.05
Perforation Size			
Small	13	13	p>0.05
Medium	14	9	
Large	11	11	
Side of Ear			
Left	20	15	p>0.05
Right	18	18	
Ear Discharge			
Dry	32	30	p>0.05
Wet	6	3	
Duration (days)	2.9 ± 1.8	2.7 ± 1.5	p>0.05
Healing Rate (%)	84.8	94.7	p>0.05

## Results

A total of 79 patients who were treated for TMP were analyzed. Three cases from the spontaneous closure group and five cases from the paper patch group were excluded from the study because their follow-ups were incomplete. Thus, a total of 71 patients were included in this study, with 38 patients (17 females, 21 males) in the paper patch group and 33 patients (16 females, 17 males) in the spontaneous closure group. In total, 38 (53.5%) patients were males and 33 (46.5%) were females, and there were 18 male patients and 15 female patients in the spontaneous closure group and 20 male patients and 18 female patients in the paper patch group. The age of the patients ranged from 2 to 16 years (10.47 ± 4.38 on average). There was no statistically significant difference between the two groups in terms of age and sex (p > 0.05).

The perforations sizes were as follows: 13 cases of small perforation, 9 cases of medium perforation, and 11 cases of large perforation in the spontaneous closure group; and 13 cases of small perforation, 14 cases of medium perforation, and 11 cases of large perforation in the paper patch group. The perforation closure rates were 84.8% and 94.7% in the spontaneous closure group and the paper patch group, respectively; the difference between the two groups was not statistically significant. Although there was no statistically significant difference between the groups in terms of closure of small and medium perforations, the closure rate in large perforations was 90.9% (10/11) in the paper patch group and 63.6% (7/11) in the spontaneous closure group, and the difference was statistically significant (p < 0.05). The perforation closure rates in perforations contacting the malleus were 58.3% and 91.6% in the spontaneous closure group and the paper patch group, respectively; the difference between the two groups was statistically significant (p < 0.05). The closure results at the end of the three-month follow-up period by contact with the malleus and perforation size are presented in Table 2.

**Table 2:** The closure and average healing time results

	N	Healing Rate (%)	Average Healing Time (day)
Spontaneous Closure Group			
Size			
Small	13	12 (92.3)	13.6 ± 2.8
Medium	9	9 (100)	20.2 ± 3.2
Large	11	7 (63.6)	29.7 ± 4.1
Contact with the malleus			
Yes	12	7 (58.3)	27.6 ± 4.5
No	21	21 (100)	13.8 ± 2.1
Paper Patch Group			
Size			
Small	13	13 (100)	9.6 ± 1.8
Medium	14	13 (92.8)	9.9 ± 2.3
Large	11	10 (90.9)	19.5 ± 4.8
Contact with the malleus			
Yes	12	11 (91.6)	20.2 ± 3.1
No	26	25 (96.1)	8.9 ± 2.3

The difference that was found between the spontaneous closure group and the paper patch group in terms of mean recovery time independent of the perforation size and contact with the malleus was not significant (p > 0.05). However, it was revealed that the duration of perforation closure increased with the increase in perforation size and contact with the malleus. Independent of the groups, the mean recovery times were 11.6, 15.1, and 24.6 days for small, medium, and large perforations, respectively. When the groups were considered as a whole, the recovery time for the perforations contacting the malleus was 23.9 days and that for the perforations not contacting the malleus was 11.3 days.

A total of 38 cases of perforations were caused by direct trauma (53.5%), whereas 33 were caused by blunt trauma 33 (46.5%). The most common causes of direct trauma included the use of cotton buds in 29 (76.3%) cases, sharp and pointed toy pieces in 7 (18.4%) cases, and use of toothpick in 2 (5.3%) cases. The most common causes of blunt trauma included slapping in 25 (75.7%) cases, falling in 3 (9.1%) cases, being hit by a ball in 3 (9.1%) cases, and barotrauma in 2 (6.1%) cases. There was no statistically significant difference between the groups in terms of causes of trauma (p > 0.05).

## Discussion

The tympanic membrane is a thin structure of 0.1 mm thickness and 81 mm^2^ area that separates the external auditory canal from the middle ear, and it conveys sound to middle ear ossicles [7]. Patients may have complaints such as pain, bloody discharge from the ear, and decreased hearing if perforation develops. Perforation of the tympanic membrane causes loss of hearing by distorting impedance matching and impeding the pressure differential created by sounds, which finally results in a mismatch in the ossicular link [8]. Various factors such as perforation size, middle ear volume and size, and frequency of hearing test affect the loss of hearing that occurs [8]. In many studies in the literature, various materials such as Gelfoam (Pfizer, New York, NY, USA), autologous fat, paper, and fibrous glue have been used to close the perforations of the tympanic membrane [5,6]. However, to the best of our knowledge, there are no studies that compared spontaneous closure and surgical interventions in TMP in pediatric cases.

Previous studies report closure rates of 85.9-97% in TMP [9,10]. Although there are limited data on pediatric patients, in this study, the spontaneous closure rate in children was determined to be 84.8%, which was in parallel with the literature considering the available information. This high rate of spontaneous closure rate may encourage clinicians to go for clinical follow-up before any surgical intervention; however, the longer recovery time found in the spontaneous closure group compared with the paper patch group and the low recovery rate found in cases of large perforations and perforations contacting the malleus are negative factors for spontaneous closure follow-up.

Although there was no significant difference between the groups in small and medium perforations, the closure rate in large perforations was 90.9% in the paper patch group and 63.6% in the spontaneous closure group, and the difference between these two groups was statistically significant (p < 0.05). Considering these findings, it can be suggested that paper patch procedure is more effective in children with large perforations in their eardrum caused by trauma. Moreover, we recommend that the paper patch procedure be performed in cases where the perforation is in contact with the malleus due to the closure rates that were found to be statistically significantly higher in the paper patch group than in the spontaneous closure group.

This is the first study to investigate TMP in children included in the literature in English language. However, the main limitation of this study is that only a limited volume of data obtained from the patients’ medical histories could be analyzed owing to the retrospective nature of the study. Nevertheless, it may be inspiring for clinicians in managing such perforations in pediatric cases.

## Conclusions

Both methods have high rates of recovery in pediatric cases of traumatic membrane perforation. However, in cases where there is a large perforation or contact with the malleus, which are criteria for poor prognosis in terms of spontaneous closure, patients should be treated by performing the paper patch procedure, which is a simple and cheap method. Patients who were diagnosed with a large perforation contacting the malleus, which is an indicator of poor prognosis for spontaneous closure, in the Emergency Department should therefore be immediately referred to the otorhinolaryngology clinic. Further randomized controlled studies involving a larger patient population are needed to further clarify our results.
